# Reference role of middle turbinate axilla in lacrimal sac localization assisted by computed tomographic dacryocystography-reference value of middle turbinate in locating lacrimal sac

**DOI:** 10.1186/s12886-022-02740-0

**Published:** 2022-12-20

**Authors:** Lihong Yang, Hongxun Li, Zhi Yin, Lei Zhang, Zhenhai Yang

**Affiliations:** Tianjin Eye Hospital, Tianjin Key Laboratory of Ophthalmology and Vision Science, Affiliated Eye Hospital of NanKai University, Clinical College of Ophthalmology of Tianjin Medical University, Tianjin, 300020 China

**Keywords:** Endoscopic surgery, Dacryocystorhinostomy, CT dacryocystography, Middle turbinate axilla, Common canaliculus, Maxillary frontal process

## Abstract

**Background:**

The middle turbinate axilla (MTA) has always been used as a stable anatomic landmark for endoscopic surgeons to locate the lacrimal sac on the lateral nasal wall. Yet, little is known about whether the lacrimal sac size will affect the positioning effect of MTA on lacrimal sac. The aim of this study was to investigate the regularity of lacrimal sac size and lacrimal sac localization through the reference position of the MTA on computed tomographic dacryocystography (CT-DCG) images.

**Methods:**

A series of 192 endoscopic dacryocystorhinostomy (DCR) surgeries were performed. All the patients had been diagnosed as unilateral nasolacrimal duct obstruction and received CT-DCG examinations. According to the maximum transverse diameter of the lacrimal sac on CT-DCG, the patients were classified into three groups. Measurements were taken on CT-DCG parasagittal images.

**Results:**

The average distance from the sac superior fundus (SSF) to the MTA was 7.52 mm ± 3.23 mm, and it increased with the increase of the maximum transverse diameter of the sac among groups (*p* < 0.01). The average distance from the common canaliculus (CC) to the MTA was 3.95 mm ± 2.49 mm. No significant difference was observed among the groups (*p* = 0.11). The average distance from the CC to the SSF was 3.41 mm ± 1.31 mm, and it increased with the increase of the sac transverse diameter among groups (*p* < 0.01).

**Conclusions:**

The lacrimal sac can be accurately located on the lateral nasal wall by the reference position of the MTA on CT-DCG images. The distance of the SSF to the MTA and the SSF to the CC is related to the lacrimal sac size. The relative position of the CC to the MTA is relatively stable on CT-DCG images, which make it possible to locate the lacrimal sac of different sizes and the corresponding nasal mucosa incision design in endoscopic DCR.

## Background

Endoscopic dacryocystorhinostomy (DCR) surgery is an effective treatment for managing nasolacrimal duct obstruction and dacryocystitis by reconstructing the lacrimal drainage system (LDS) of nasal [1]. In previous studies, the high success rate of endoscopic DCR ranged from 80 to 95%. However, there was still 4% to 13% surgical failure occurred [2, 3]. The main reasons for failure were associated with inappropriate position of the ostium, extra damage of mucosa around the lacrimal sac, inadequate exposure and incomplete marsupialization of the lacrimal sac [4]. Accurate positioning of the lacrimal sac is contributing to the formation of ideal DCR ostium, which is the key to improving the success rate of DCR and achieving effective lacrimal drainage.

Computed tomographic dacryocystography (CT-DCG) can be used to locate the lacrimal sac, and it is proven to be a safe, objective, non-invasive and reliable diagnostic method for assessing the nasolacrimal duct system in patients prior to DCR. The sac superior fundus (SSF) and the common canaliculus (CC) are the main signs of the lacrimal sac on CT-DCG. The middle turbinate axilla (MTA) has always been used as a stable anatomic landmark for endoscopic surgeons to locate the lacrimal sac on the lateral nasal wall. And the position of the lacrimal sac is commonly found to be anterior to the MTA, and two-thirds of the lacrimal sac length are superior to the insertion of the middle turbinate on the lateral nasal wall [5]. In previous CT-DCG study, the SSF is shown extending 8-10 mm above the middle turbinate insertion, and the height of the sac above the common canalicular opening is approximately 5 mm [6]. However, does this sac location theory apply to everyone? For different size of the sac, does the distance of the SSF to the MTA remain stable? How about the distance of the CC to the MTA? By far, little is known about whether the lacrimal sac size will affect the positioning effect of MTA on lacrimal sac. In this retrospective study, the average distance from the SSF and the CC to the MTA was studied on CT-DCG Images in groups, aiming to investigate the availability of locating the lacrimal sac of different size by the reference position of the middle turbinate axilla. To our knowledge, there have been few related study reported before.

## Methods

### Study setting, design and population

The retrospective study was performed in accordance with the Declaration requirement of Helsinki for research involving human subjects. The protocol and waiver of informed consent used in this study were approved by the Ethics Committee of Tianjin Eye Hospital.

Consecutive endoscopic DCR patients from January 2018 to December 2020 were included in the study. All the patients were Chinese from Asia. Medical records including clinical information and surgical notes and videos were reviewed. A total of 192 eyes were included in the study and underwent CT-DCG and subsequent endoscopic DCRs. Lacrimal irrigation was used as a standard preoperative evaluation on the patency and reflux of the lacrimal drainage system. Nasal endoscopic exam was used to determine the necessity of additional nasal surgery (septoplasty, middle turbinoplasty or sinus surgery) preoperatively. All the patients included in the study had been diagnosed as nasolacrimal duct obstruction. Of all, 130 patients were diagnosed with chronic dacryocystitis and 62 patients were diagnosed with simple nasolacrimal duct obstruction without purulent discharge. The exclusion criteria were history of facial bone fracture, previous lacrimal surgery, obstruction of lacrimal canaliculus, lacrimal canaliculi inflammation and children. Patients who had acute dacryocystitis process or a history, mucocoele and suspected lacrimal drainage tumor were also excluded. Patients who underwent additional nasal surgery were included in the analysis.

### CTDCG acquisition and grouping

In order to discharge purulent secretion, all the patients underwent lacrimal irrigation with saline before performing DCG. And 0.5–1.0 ml of water-soluble contrast medium (Iohexol, 755 mg/ml) was slowly injected into the examined LDS from inferior as well as superior punctum. Axial view CT scan with a thickness of 1 mm was performed with soft-tissue and bone windows. And axial images data were reconstructed into coronal and parasagittal images along the main axis of the LDS for subsequent measurements. Measurements on the CT images were performed using software on a GE Advantage Windows workstation. All DCG images were evaluated by two experienced radiologists, and the final agreement was reached by consensus.

According to the maximum transverse diameter of the lacrimal sac on CT-DCG, the patients were classified into three groups. 1) large lacrimal sac (LLS), the maximum sac transverse diameter > 5 mm; 2) medium lacrimal sac (MLS), the maximum sac transverse diameter was2-5 mm; 3) small lacrimal sac (SLS), the maximum sac transverse diameter < 2 mm.

### CTDCG processing

**Fig. 1:**
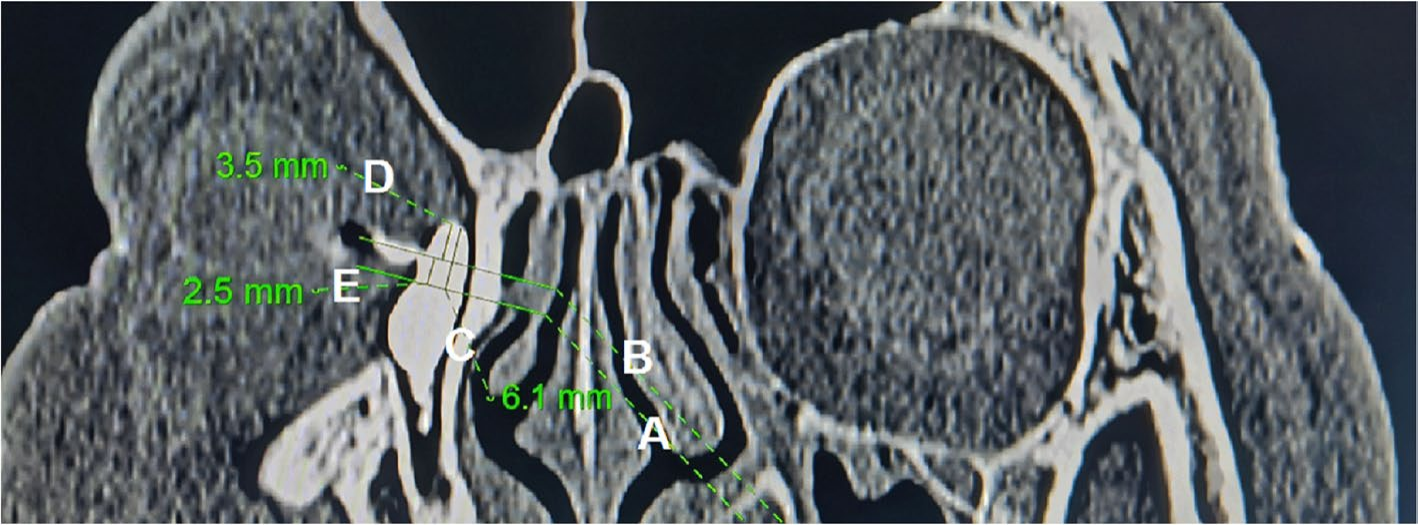
Measurements in the long axis of a large lacrimal sac on computed tomographic dacryocystography (CT-DCG) parasagittal images. **A**, a line drew from the middle turbinate axilla (MTA) to the long axis of the sac at right angles. **B**, a line drawn through the common canaliculus (CC) to the long axis of the sac at right angles. **C**, the height of the sac above the MTA. **D**, the height of the sac above the CC. **E**, the distance from the MTA to the CC

**Fig. 2:**
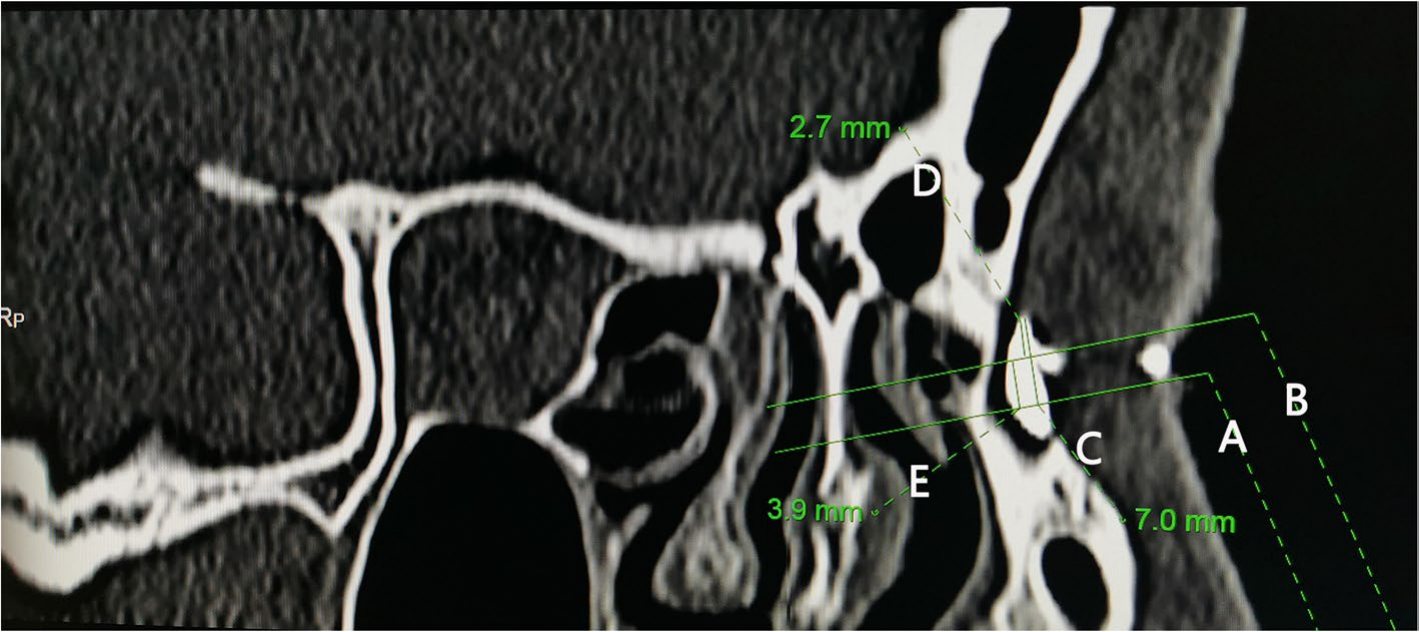
Measurements in the long axis of a medium lacrimal sac on computed tomographic dacryocystography (CT-DCG) parasagittal images. **A**, a line drew from the middle turbinate axilla (MTA) to the long axis of the sac at right angles. **B**, a line drawn through the common canaliculus (CC) to the long axis of the sac at right angles. **C**, the height of the sac above the MTA. **D**, the height of the sac above the CC. **E**, the distance from the MTA to the CC

**Fig. 3:**
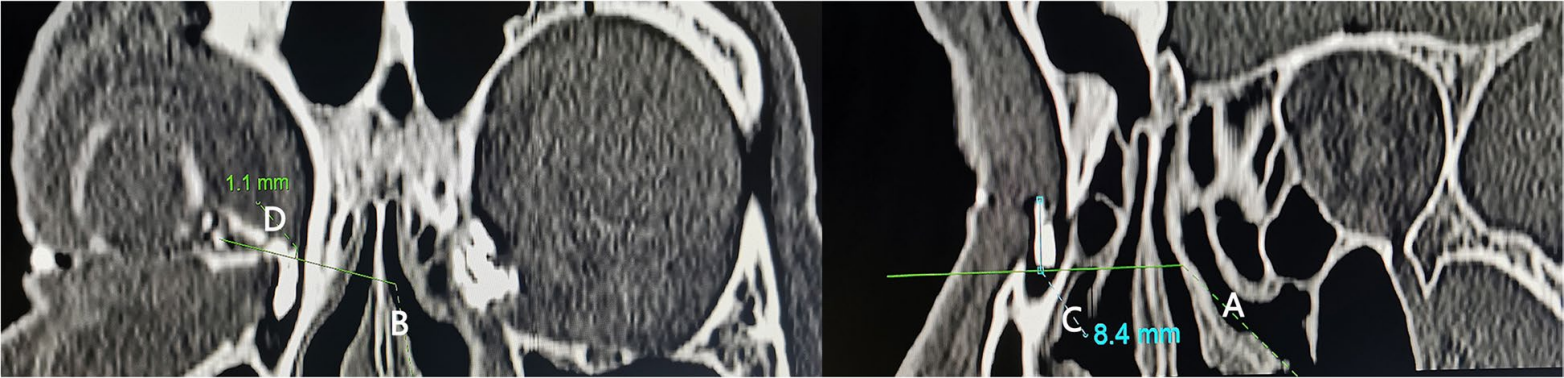
Measurements in the long axis of a small lacrimal sac on computed tomographic dacryocystography (CT-DCG) parasagittal images. For most patients, it is difficult to display the common canaliculus (CC) and the middle turbinate axilla (MTA) simultaneously on coronal CT-DCG images, and it is difficult to measure their distance directly. In this study, the distance from the MTA to the CC was calculated by the difference between the height from the SSF to the MTA and the height of the sac above the CC. **A**, a line drew from the middle turbinate axilla (MTA) to the long axis of the sac at right angles. **B**, a line drawn through the common canaliculus (CC) to the long axis of the sac at right angles. **C**, the height of the sac above the MTA. **D**, the height of the sac above the CC

All measurements below were taken from the long axis of the lacrimal sac in three groups respectively (see Figs. 1, 2 and 3). According to Wormald measuring method [5], a line was drawn from the MTA to the long axis of the sac at right angles, which was used to measure the height of the sac above the middle turbinate. Another line was drawn through the CC at right angles to the sac long axis, which was used to measure the height of the sac above the CC. And the distance from the MTA to the CC was calculated by the difference between the above two height. Meanwhile, the thickness of the maxillary frontal process (MFP) was measured on the line drawn through the CC. In order to reduce the measurement error, the CTDCG images were reviewed by two radiologists who were blind to the experimental design and grouping, and all the measurements were taken three times respectively and then averaged.

**Fig. 4:**
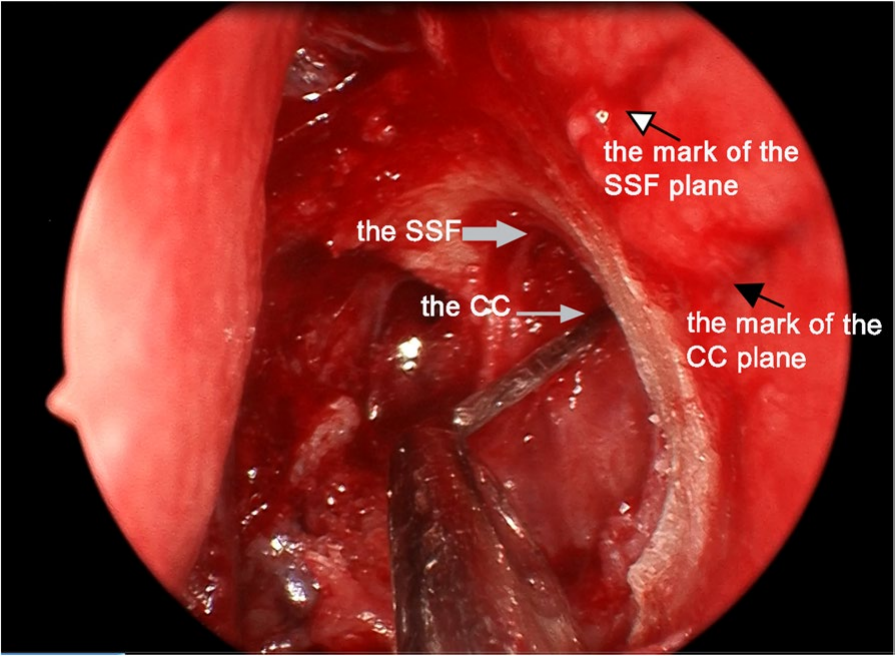
Accuracy verification of preoperative lacrimal sac location. During surgery, marks made with a marker on the lateral nasal wall can become shallow or even disappear. Before operation, we marked the planes of the CC and the SSF by making two shallow mucosa incisions (indicated by the black arrow and the white arrow with black edge) which were at right angles to the sac long axis on the lateral nasal wall. When the marsupialization of the sac was completed and the SSF was exposed, the accuracy of preoperative marks can be verified by the relationship between the shallow incision extension lines and the position of the CC and SSF

**Fig. 5:**
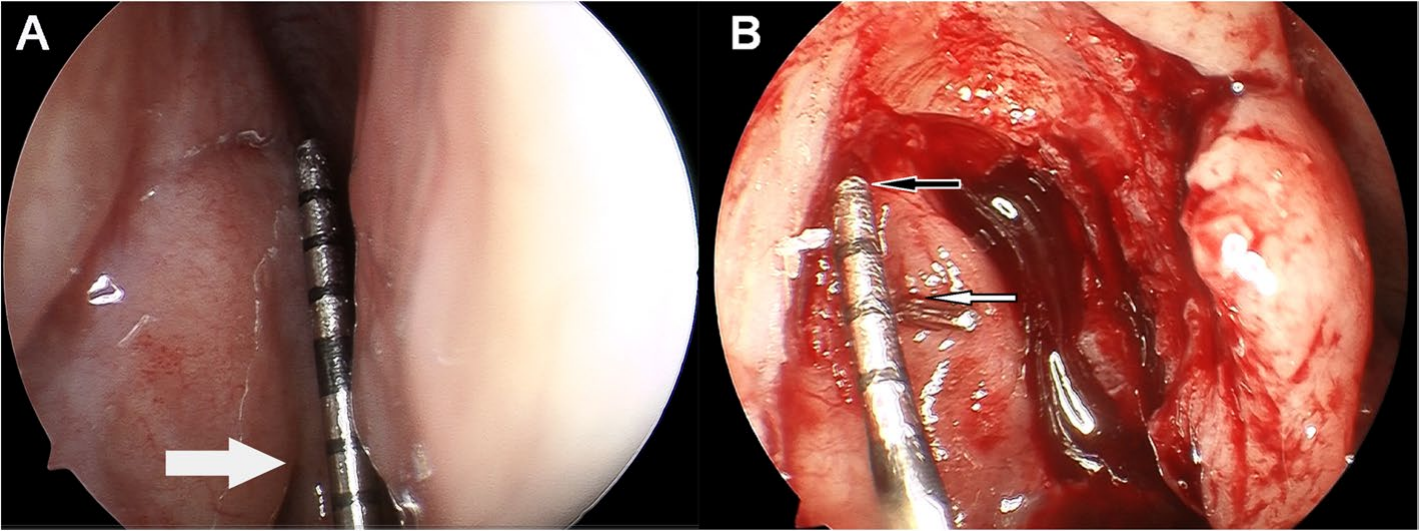
Intraoperative view of endoscopic dacryocystorhinostomy (endoscopic DCR). **A**, the sac location on the lateral nasal wall with graduating probe. The white arrow shows the middle turbinate axilla (MTA). **B**, The black arrow indicates the exposed sac superior fundus (SSF) and the white arrow shows the opening of the common canaliculus (CC). Intraoperative measurements were performed

According to the reference position of the middle turbinate insertion on CT-DCG, the plane of the CC and that of the SSF were marked on the lateral nasal wall with graduated probe before operation, which were used to compare with the actual position of the CC and the SSF during operation (see Fig. 4). Furthermore, the surgical incision of nasal mucosa was designed. All patients were performed with standardized endoscopic DCR technique which was described by Wormald previously [7]. The SSF and the opening of the CC were exposed. And the intraoperative measurements were performed, including the height of the SSF to the CC and the SSF to the MTA (see Fig. 5). Surgical outcome was not the focus of discussion, so there was no further success rate analysis in this study.

### Statistical analysis

The results of continuous variables were presented as mean ± standard deviation (SD), whereas that of categorical variables were presented as percentages. Differences in study groups were compared in terms of mean age, the distance between the SSF, the CC and the MTA using the one-way analysis of variance (ANOVA). LSD Post hoc test was used for multiple comparison after it. Chi-square or Fisher’s exact test was used to compare proportions of gender, side, poor imaging LDSs eyes, and combined additional nasal surgery. Data were analysed with IBM SPSS 24 statistical software. All tests were two-sided, and *p* < 0.05 was considered as statistically significant.

## Result

One hundred and ninety two consecutive patients, 140 women and 52 men, mean age 52.8 years (range22-83), were recruited to the study. All the patients had been diagnosed as unilateral nasolacrimal duct obstruction. CT-DCG was performed on affected eyes in patients. No CT-DCG related side effects occurred. All lacrimal sac images can be detected on CT-DCG. Nevertheless, images of the lacrimal canaliculi or the CC in 12 eyes were not clearly displayed and were excluded. In 53 eyes, the images of the canaliculi or the CC that were thin but did not affect subsequent image measurements were included in the analysis. Demographic data and preoperative findings are summarized in Table 1. All patients underwent endoscopic DCRs, and 17 DCRs underwent endoscopic DCR combined with additional nasal surgery (see Table 2). There was no statistically significant difference in mean age, gender, side, poor imaging LDSs cases, combined with additional nasal surgery between study groups.

**Table 1:** Comparison of groups’ demographic data and preoperative findings

Characteristics	LLS group	MLS group	SLS group	*p*-value
Eyes	59	65	68	
Age (y ± SD)	50.7 ± 14.3	51.8 ± 12.7	56.7 ± 10.8	0.12^a^
Gender n (%):				0.77^b^
Male	14 (23.73)	19(29.23)	19 (27.94)	
Female	45 (76.27)	46 (70.77)	49 (72.06)	
Side eyes (%):				0.73^b^
Left	28 (47.46)	34 (52.31)	31 (45.59)	
Right	31 (52.54)	31 (47.69)	37 (54.41)	
Poor imaging LDSs eyes (%):	5 (8.47)	3 (4.62)	4 (5.89)	0.50^c^
Lacrimal canaliculi	1 (1.69)	2 (3.08)	2 (2.94)	
Common canaliculus	4 (6.78)	1 (1.54)	2 (2.94)	

Values are presented as mean ± standard deviation or number (%)

*LLS* (Large lacrimal sac), *MLS* (Medium lacrimal sac), *SLS* (Small lacrimal sac)

^a^One- Way ANOVA; ^b^Chi-square test; ^c^Fisher’s exact test. *Significance p* < *0.05*

**Table 2:** The distribution of primary dacryocystorhinostomy combined with additional nasal surgery in the study groups

Characteristics	LLS group (eye = 54)	MLS group (eye = 62)	SLS group (eye = 64)	*p*-value
Primary DCR + additional nasal surgery eyes (%):	5 (9.26)	7 (11.29)	5 (7.81)	0.99^a^
Septoplasty	1 (1.85)	2 (3.23)	2 (3.13)	
Middle turbinoplasty	3 (5.56)	4 (6.45)	2 (3.13)	
Sinus surgery	1 (1.85)	1 (1.61)	1 (1.56)	

Values are presented as number (%)

*LLS* (Large lacrimal sac), *MLS* (Medium lacrimal sac), *SLS* (Small lacrimal sac), *DCR* (Dacryocystorhinostomy)

^a^Fisher’s exact test. *Significance p* < *0.05*

Surgical data showed that the location of the lacrimal sac during the operation was basically consistent with the measurements of CT-DCG lacrimal sac positioning. The measurements between the SSF, the CC and the MTA in the long axis of the sac were presented in Table 3.

**Table3:** Measurements of computed tomographic dacryocystography (CT-DCG) images in study groups

Measurements on CT-DCG images(mm)	LLS group (eye = 54)	MLS group (eye = 62)	SLS group (eye = 64)	*p*-value
MTA—SSF	8.63 ± 3.05	7.59 ± 2.92	5.96 ± 2.56	0.00^a^
MTA—CC	4.25 ± 2.76	4.12 ± 2.63	3.33 ± 1.79	0.14^a^
CC—SSF	4.38 ± 1.05	3.46 ± 1.20	2.63 ± 1.15	0.00^a^
Thickness of MFP at the level of CC	3.47 ± 1.27	3.49 ± 1.28	3.56 ± 1.13	0.94^a^

Values are presented as mean ± standard deviation

*CT-DCG* (Computed tomographic dacryocystography), *LLS* (Large lacrimal sac), *MLS* (Medium lacrimal sac), *SLS* (Small lacrimal sac), *MTA* (Middle turbinate axilla), *SSF* (Sac superior fundus), *CC* (Common canaliculus), *MFP* (Maxillary frontal process)

^a^One- Way ANOVA; *Significance p* < *0.05*

**Fig. 6:**
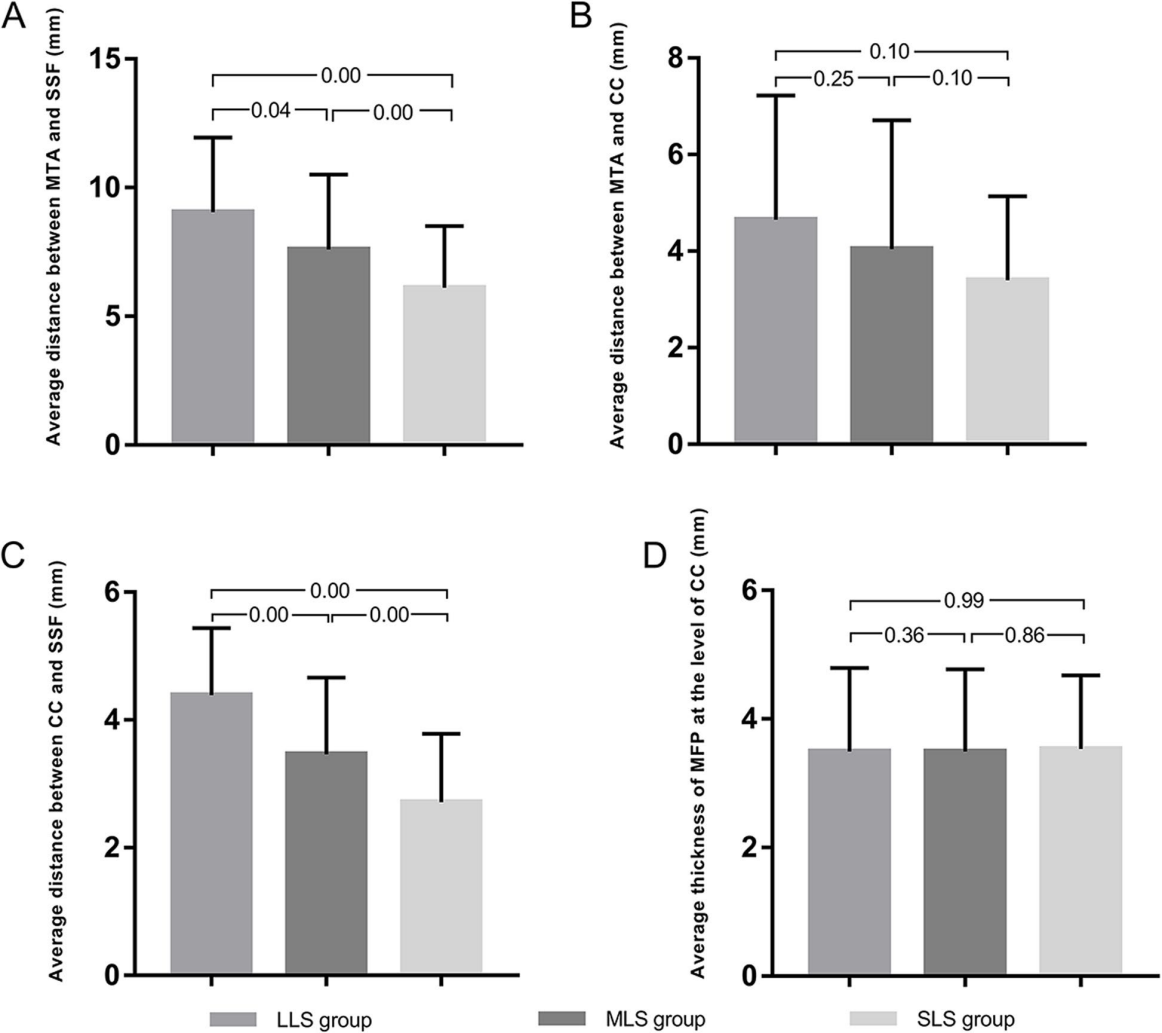
Comparison of measurements on computed tomographic dacryocystography (CT-DCG) images between groups. **A**, Average distance between the middle turbinate axilla (MTA) and the sac superior fundus (SSF); **B**, Average distance between the MTA and the common canaliculus (CC); **C**, Average distance between the CC and the SSF; **D**, Average thickness of maxillary frontal process (MFP) at the level of the CC. Data is presented as mean ± standard deviation. Numbers above the bars represent the *p* values. Abbreviations: LLS, large lacrimal sac; MLS, medium lacrimal sac; SLS, small lacrimal sac

Of all patients, the SSF was located above the MTA on CT-DCG images, and the average distance from the SSF to the MTA was 7.52 mm ± 3.23 mm. According to the aforementioned grouping, the average distance from the SSF to the MTA increased with the increase of the maximum transverse diameter of the sac in groups. There was a significant difference between groups (*p* < 0.01) (see Fig. 6A).

Of all 180 eyes, the CC of 6 eyes (3.33%) were located below the MTA, and that of 174 eyes (96.7%) were above. The average distance from the CC to the MTA was 3.95 mm ± 2.49 mm. There was no significant difference among the groups (*p* = 0.11) (see Fig. 6B).

In all cases, the average distance from the CC to the SSF was 3.41 mm ± 1.31 mm. Moreover, the average distance from the CC to the SSF increased with the increase of the maximum transverse diameter of the sac in groups. The difference among groups was statistically significant (*p* < 0.01) (see Fig. 6C).

The thickness of the MFP at the level of the CC was measured. And the average thickness was 3.50 mm ± 1.24 mm. There was no significant difference between groups (*p* = 0.70) (see Fig. 6D).

## Discussion

Most of the reasons for failure of endoscopic DCR can be attributed to a poor understanding of endonasal anatomy and the lacrimal sac position on the lateral nasal wall, which can lead to a wrong location of the rhinostomy [8]. Different from the procedure of external approach, the first step of endonasal DCR is to determine the projection position of the lacrimal sac on the lateral nasal wall. In order to locate the lacrimal sac accurately, it is necessary to utilize a stable surgical reference mark and determine its relative position with the lacrimal sac on the lateral nasal wall.

The axilla of the middle turbinate and the maxillary line are the major landmarks utilized by endonasal surgeons to localize the lacrimal sac. In addition,the lacrimal sac is adjacent to the maxillary frontal process(FPM), the lacrimal bone, agger nasi air cell and the uncinate process [9–13].The axilla of the middle turbinate tends to be a constant endonasal anatomical landmark, which can be utilized to localize the lacrimal sac in DCR surgery [6]. Wormald et al.’s study on CT-DCG showed that the sac is located 8-10 mm above the axilla of the middle turbinate [5]. In other studies, the fundus of the sac is an average of 4.73 mm ± 2.86 mm, 6.6 mm ± 1.3 mm above the axilla of the middle turbinate, respectively [9, 14]. Apart from the ethnic origin, were the differences in these findings related to other factors, such as the lacrimal sac size? Previous studies have rarely addressed the above issues further.

Through the measurement of CT-DCG images, the SSF of all cases was located above the MTA in this study. And the average distance from the axilla to the sac fundus was 7.52 mm ± 3.23 mm. Our result is very close to the results of Wormald et al.’s. However, the grouped study according to the transverse diameter of the lacrimal sac showed that the average distance from the axilla to the sac fundus in the large, medium, and small lacrimal sac groups was 8.63 mm ± 3.05 mm, 7.59 mm ± 2.92 mm, 5.96 mm ± 2.56 mm, respectively. With the increase of the transverse diameter of the sac, the distance from the axilla to the sac fundus gradually increased. Our results show that the size of the lacrimal sac is closely related to the relative position of the superior fundus of the sac. The relative position of the lacrimal sac fundus is not constant. Anatomically, the lacrimal sac is located in the lacrimal sac fossa composed of the frontal process of the maxillary bone and the lacrimal bone. The medial and anterior inferior part of the lacrimal sac is surrounded by bony structures of the lacrimal sac fossa. When chronic inflammation occurs, due to the continuous secretion of the mucosal epithelium of the lacrimal sac, the accumulation of purulent secretions leads to the increase of pressure in the lacrimal sac. Different from acute dacryocystitis, this slowly increasing pressure will cause the bone of the lacrimal sac fosa to compress and form a depression, which will expand the lacrimal sac to both sides and above, resulting in the increase of the transverse diameter of the lacrimal sac, and the corresponding increase of the distance between SSF and MTA. It is speculated that this may be why the size of the lacrimal sac is closely related to the relative position of the SSF.

In our study, the CC of most patients is above the MTA, and the average distance from the CC to the axilla is 3.95 mm ± 2.49 mm. Moreover, there was no statistically significant difference between groups. These results indicate that the position of the CC relative to the axilla is relatively stable and is less related to the size of the lacrimal sac. Because the CC is interwoven and surrounded by hard and thick tissues such as muscle fibers and medial canthal ligaments, it is speculated that the relatively stable position of the CC may be related to these anatomical factors [15]. One of the main reasons given for the failure of external DCR surgery is inadequate bone removal in the sac projection area, which may also be an important factor for the failure of endoscopic DCR surgery [16]. The CC provides a valuable landmark for endoscopic surgeons [17]. If the CC is visible through the open sac, the surgeon can be reassured that the bone removal is sufficiently high and most of the sac is exposed. In ideal DCR procedure, the area of bony resection around the CC should be at least 3–5 mm in diameter [18]. If the level of the CC is determined on the lateral nasal wall during the DCR operation, the ideal position of nasal mucosal incision and bone resection can be obtained by referring to it. Our study shows that it is feasible to determine the level of the CC on the lateral nasal wall through the MTA, which is of great significance for accurate localization of the lacrimal sac of different sizes and full opening of the lacrimal sac cavity in DCR surgery. There are few reports about the location of the CC relative to the MTA. The reason may be related to the difficulty in displaying the CC and the MTA on a coronal CT-DCG image at the same time, and the difficulty in directly measuring their distance. In this study, the distance from the axilla to the CC was obtained by calculating the difference between the distance from the SSF to the axilla and the distance from the SSF to the CC. To decrease the measuring error, all the measurements of CTDCG images were taken three times by two radiologists respectively and then averaged. It is expected that there will be better measurement methods to further verify the positioning of the CC in the future.

In our study, the average distance from the fundus of the sac to the CC was 3.41 mm ± 1.31 mm. With the increase of the transverse diameter of the sac, the distance from the fundus of the sac to the CC gradually increased. Our results were very close to the in-vivo measurements reported by Singh et al., which also found a higher SSF location in the enlarged lacrimal sac[19]. However, Wormald et al.used CT-DCG to show that the fundus of the sac lies about 5 mm above the common canalicular opening. The reason for the difference between the studies may be related to the ethnic origin of the research subjects [14, 20]. In addition, since previous studies seldom grouped according to the size of the sac, the composition ratio of the sac size may also be a factor leading to the differences.

In view of the importance of fully exposing the common canalicular opening in the sac during endoscopic DCR surgery, the MFP at the level of the CC should be removed during the operation. We measured the thickness of the MFP at the level of the CC, and found that the bone thickness was on average 3.50 mm ± 1.24 mm, which had no significant correlation with the size of the sac. This is very close to the results of previous studies in which the bone thickness of the MFP was 3-6 mm above the maxillary line [6, 14]. In the process of powered endoscopic DCR, the tip of the grinding drill is spherical, it shapes the bone of the MFP into a curve plane between the SSF and the nasal mucosal incision. The thicker the bone of the MFP, the higher the height of the curved bone, and the height is usually 1-2 mm. Therefore, in order to fully open the lacrimal sac and reach the target height, the nasal mucosa incision should be designed to add an additional 1-2 mm incision height.

In the routine DCR procedure, the design of the nasal mucosal incision should aim at fully exposing the SSF and making the complete marsupialization of the sac. In our study, the position of the CC relative to the axilla is relatively stable and is less related to the size of the lacrimal sac. The average distance from the axilla to the CC is about 4 mm. For patients with large and medium lacrimal sacs, the lacrimal sacs should be exposed at least 3 mm above the CC to achieve complete marsupialization of the SSF [19]. And the height of the MFP curved bone from the SSF to the nasal mucosal incision should be added by 1 to 2 mm. It is estimated that the position 8-9 mm above the axilla can be designed as the height of the nasal mucosa incision of the endoscopic DCR for patients with large and medium lacrimal sac. This can served as a reference to simplify the lacrimal sac localization process in these patients.

Small lacrimal sac DCR surgery has its uniqueness, and the success rate of surgery is not ideal [21, 22]. The particularity of the small lacrimal sac DCR operation lies in a narrow cavity and less lacrimal sac mucosal available which makes it more difficult to fully marsupialize and difficulty in exposing the sac. In small lacrimal sac DCR surgery, more attention should be paid to reducing nasal mucosa damage and bone exposure over the location of the ostium [23, 24]. In the small sac DCR operation, the large mucosal incision made in conventional DCR surgery may not be suitable. The larger the mucosal excision, the more mucosal damage and the bare bone, which increases the risk of ostium granulomas proliferation and cicatricial closure [25]. In our study, the distance of the SSF to the CC varies from a minimum of 0.6 mm to a maximum of 4.2 mm in the small sac group. Referring to the level of the CC and the distance from the SSF to the CC, personalized nasal mucosal incision can be designed for patients with small lacrimal sac. And it is of great significance to improve the success rate of small lacrimal sac DCR surgery.

In conclusion, our study shows that it is feasible to locate the sac through the relative position of the CC and the MTA on CT-DCG images,with reference to the size of the lacrimal sac. Accurately positioning the lacrimal sac and upper nasal mucosal incision is beneficial to expose the superior most aspect of the lacrimal sac and make the complete marsupialization of the sac more feasible in all endoscopic DCR surgery. At the same time, it can avoids much more mucosal incisions than necessary during the operation, which can shorten the operation time, reduce the surgical trauma, and make the endoscopic DCR operation more safer, more efficient, and less invasive. The above is of great significance in improving the success rate of endoscopic DCR surgery. Since this study only discussed the availability of locating the lacrimal sac by CT-DCG, surgical data were limited. More locating information of the lacrimal sac and the nasal mucosa incision in endoscope DCR surgery is needed to further supplement and verify the clinical application of the above results.

## Data Availability

The datasets used and/or analysed during the current study are available from the corresponding author on reasonable request.

## Abbreviations

MTA: The middle turbinate axilla
CT-DCG: Computed tomographic dacryocystography
DCR: Dacryocystorhinostomy
SSF: Sac superior fundus
CC: Common canaliculus
LDS: Lacrimal drainage system
LLS: Large lacrimal sac
MLS: Medium lacrimal sac
SLS: Small lacrimal sac
MFP: Maxillary frontal process
